# Thermal shock resistance of various two-dimensional materials: a comparative analysis

**DOI:** 10.1039/d5ra09164k

**Published:** 2026-03-23

**Authors:** Ali Ghavipanjeh, Sadegh Sadeghzadeh, Nader Malih

**Affiliations:** a Research Assistant in Nanotechnology Engineering, Smart Micro/Nano Electromechanical Systems (SMNEMS) Laboratory, School of Advanced Technologies, Iran University of Science and Technology Tehran Iran; b Associate Professor of Nanotechnology Engineering, Smart Micro/Nano Electromechanical Systems (SMNEMS) Laboratory, School of Advanced Technologies, Iran University of Science and Technology Tehran 16846-13114 Iran sadeghzadeh@iust.ac.ir; c Department of Physics, Faculty of Science, University of Kurdistan 66177-15175 Sanandaj Kurdistan Iran

## Abstract

Understanding the ultrafast thermomechanical response of two-dimensional (2D) materials is crucial for their integration into next-generation nanoelectronic and thermal management technologies. In this study, molecular dynamics simulations are used to systematically evaluate the thermal shock resistance of graphene, C_3_N, BC_3_, three BC_6_N configurations, biphenylene, borophene, and hexagonal boron nitride (h-BN). A localised, rapid temperature increase is applied to generate stress waves, enabling direct assessment of wave-propagation velocity, decay rate, energy dissipation, atomic displacement, and temperature changes in both armchair and zigzag orientations. The findings reveal strong correlations between lattice topology and non-equilibrium thermomechanical behaviour. Graphene and C_3_N show exceptional shock tolerance, with high wave speeds and minimal attenuation; C_3_N demonstrates nearly undamped propagation. Borophene exhibits notable anisotropy, with wave transmission and dissipation depending on direction, whereas biphenylene and BC_3_ exhibit significant damping and stress irregularities due to their structures. The trends highlight how thermal shock resistance depends on bonding structure, coordination, and lattice symmetry. This work provides a unified benchmark for transient thermal-mechanical performance in 2D materials and offers valuable guidance for selecting and designing atomically thin systems for high-thermal-demand applications.

## Abstract 2

Understanding the ultrafast thermomechanical response of two-dimensional (2D) materials is crucial for their integration into next-generation nanoelectronic and thermal management technologies.

## Graphical abstract

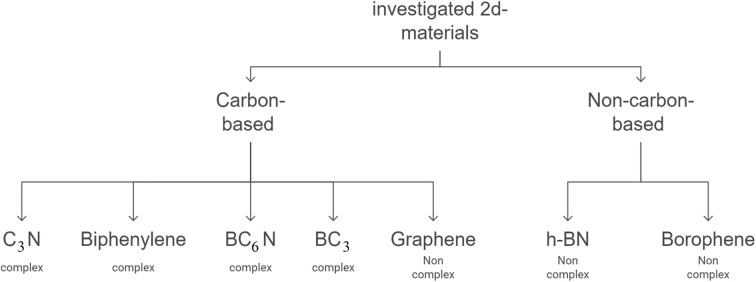

## 1 Introduction

In recent years, two-dimensional (2D) materials have garnered considerable attention due to their remarkable physical, chemical, and electronic properties.^1^ Defined by their atomically thin layered structures,^2^ these materials demonstrate exceptional mechanical flexibility,^3^ high electrical conductivity,^4^ wide surface area,^1^ and tailored band gaps.^5^ The discovery of graphene, the first and best-studied 2D material, has inspired the investigation of other 2D materials, including BC_3_, BC_6_N, C_3_N, boron nitride (BN), biphenylene, and borophene. Each of these materials exhibits unique structural and electronic properties, making them suitable for a broad array of advanced technological applications.^1,6,7^ Their atomic composition and bonding configurations primarily influence the properties and characteristics of 2D materials.^2,7^ The unique attributes of these materials stem from their dimensionality, strong in-plane bonding, and weak van der Waals interlayer interactions.^2,3,6^

2D materials offer significant applications due to their unique characteristics, which address particular technical challenges.^7^ Graphene, a monolayer of sp^2^-hybridized carbon atoms arranged in a honeycomb structure,^8^ is known for its remarkable electrical,^8^ mechanical,^9^ and thermal properties.^10,11^ Biphenylene, an atypical carbon allotrope, possesses a unique electrical structure that makes it advantageous for advanced optoelectronic devices.^12–14^ Borophene, a monolayer composed of boron atoms, exhibits metallic conductivity and anisotropic mechanical properties, rendering it ideal for flexible, high-performance electronic devices.^15–17^ Hexagonal boron nitride (h-BN) is a two-dimensional insulating material with high heat stability and mechanical strength.^18,19^ It serves as an excellent dielectric layer and high-temperature barrier, often combined with graphene in composite materials to enhance durability and heat resistance.^20–23^ Materials such as BC_3_, BC_6_N, and C_3_N, consisting of boron, carbon, and nitrogen, exhibit tailored band gaps and chemical reactivity, which are advantageous for catalysis and energy applications.^10,24–28^

**Fig. 1:**
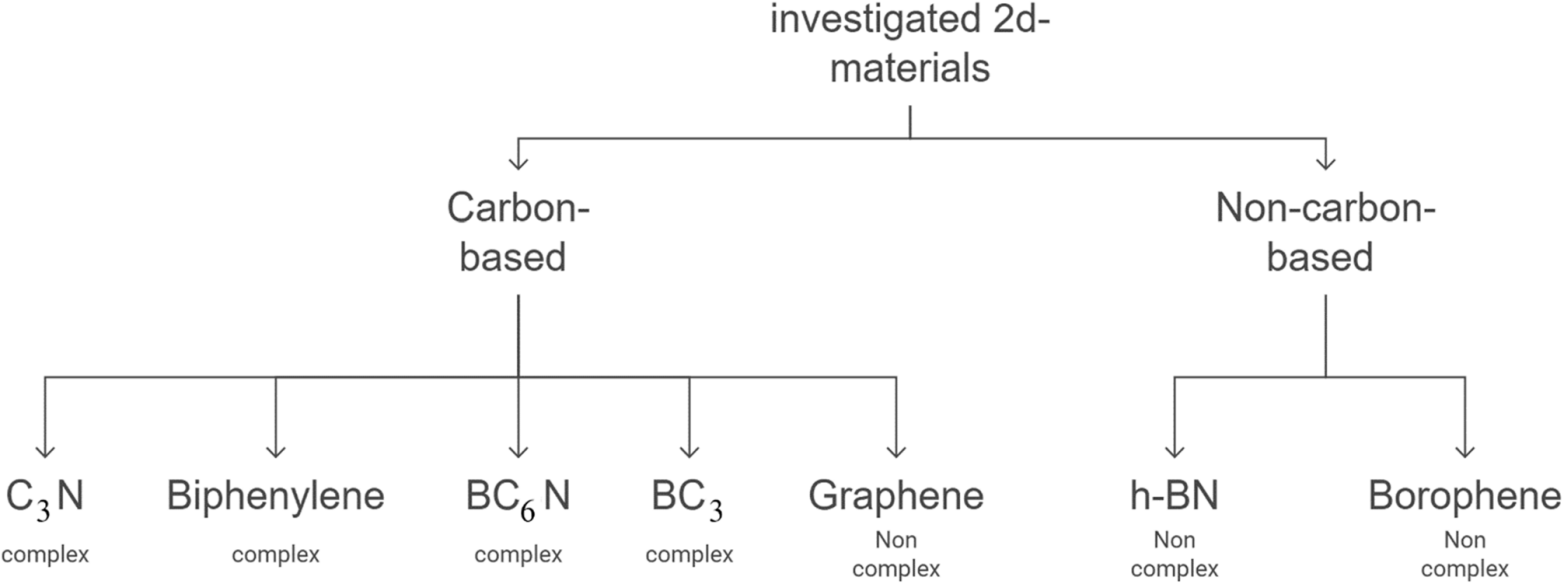
The classification of the investigated 2D materials in this work, based on their structural complexity and elemental composition.

2D materials are often categorized by structural complexity and elemental composition, particularly carbon content. For instance, graphene is classified as a non-complex, carbon-based material. In contrast, complex carbon-based systems include C_3_N, BC_3_, BC_6_N, and biphenylene. Additionally, non-carbon-based 2D materials, such as h-BN and borophene, are typically regarded as non-complex due to their simple atomic arrangements.^1,7,29^Fig. 1 illustrates this classification.

Thermal shock, caused by rapid temperature fluctuations, poses a significant challenge to the strength of two-dimensional (2D) materials, particularly in applications such as flexible electronics, energy storage systems, and sensors.^30–32^ Despite their exceptional properties, sudden thermal gradients can lead to localised stress concentrations, which may cause defects, delamination, or even catastrophic failure.^30^ Therefore, understanding how 2D materials respond to such extreme thermal conditions is essential for ensuring their reliability and durability in real-world applications.

Molecular dynamics (MD) simulations are crucial for investigating thermal shock in 2D materials at the atomic level, enabling precise control over thermal loading and real-time observation of atomic motions, stress evolution, and energy dissipation to obtain properties and mechanisms by monitoring stress, temperature, and atomic displacement, especially under extreme thermal conditions.^30,33,34^ Typically, in a shock simulation, the structure is equilibrated at a specified temperature, followed by a sudden thermal change that induces thermal stress waves. These waves evolve from compression to alternating compressive–tensile regions, reflecting non-uniform temperature and displacement fields.^34–37^

This approach aligns with previous research on stress-wave behaviour and decay in 2D materials. For instance, studies of buckyball lattices have shown that stress waves in hexagonal close-packed systems lose impact energy exponentially, underscoring the importance of the decay rate for understanding wave dynamics in two-dimensional structures.^38^ Additionally, research on carbon phenolic materials exposed to X-ray-induced thermal shocks shows that the stress-decay rate significantly influences peak stress at various depths, underscoring the importance of decay-rate analysis in thermal-shock contexts.^39^ By examining different crystallographic orientations—namely armchair and zigzag—we evaluated the anisotropic nature of wave attenuation, which is key to understanding direction-dependent thermal shock resistance. The calculated decay rates are crucial for assessing how different 2D materials or lattice orientations affect stress-wave propagation under high-temperature gradients. Unlike earlier studies that primarily focused on thermal conductivity or general mechanical properties, our research captures the transient thermoelastic response and the spatial evolution of stress during rapid thermal loading. Through simulations of realistic temperature gradients and analyses of direction-dependent factors such as stress distribution, atomic displacement, and wave speed, we aim to elucidate the fundamental mechanisms underlying thermal shock resistance in 2D materials. This knowledge is vital for improving the thermal reliability of nanoscale devices, where anisotropic properties and rapid thermal fluctuations can significantly affect performance and failure modes. Our results provide valuable insights for designing thermally robust 2D materials suited for high-performance applications. Our study bridges this gap by employing a unified MD framework to analyze various 2D materials—including graphene, C_3_N, BC_3_, three forms of BC_6_N, biphenylene, borophene, and h-BN—with material-specific interatomic potentials validated against structural metrics. We measure stress-wave speed, decay rate, displacement fields, and temperature changes in both armchair and zigzag directions. This creates the first comprehensive benchmark of thermal shock resistance across different bonding types and lattice symmetries. Including anisotropic materials such as borophene, complex lattices such as biphenylene, and multi-component systems such as BC_3_, BC_6_N, and C_3_N establishes a new comparative framework for understanding how structural differences influence ultrafast thermomechanical stability. These contributions provide a solid basis for selecting materials and designing devices capable of withstanding extreme thermal transients.

## 2 Simulation methods

**Fig. 2:**
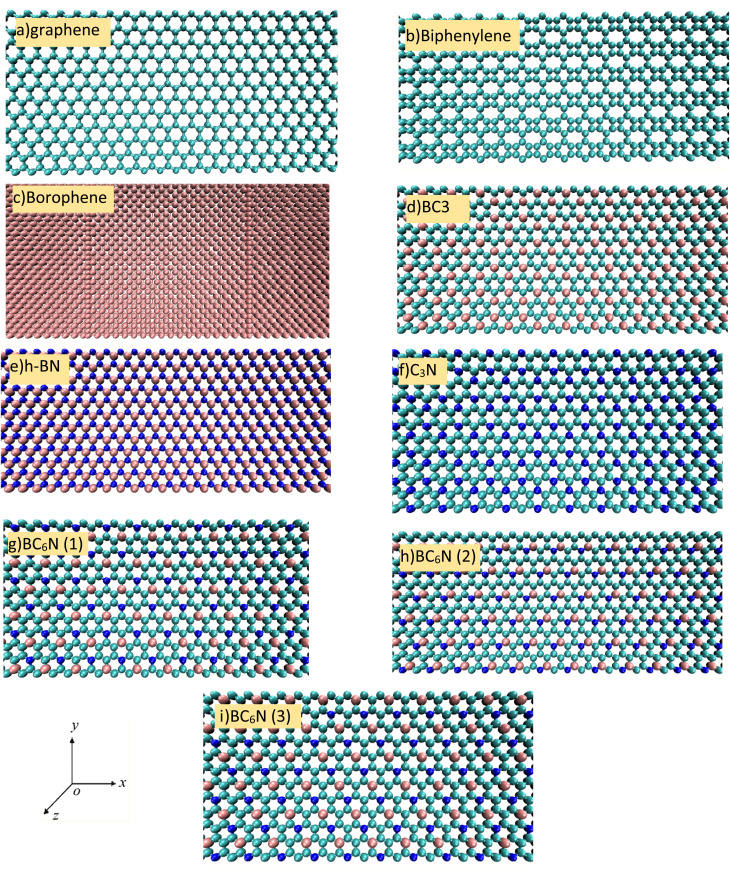
The initial structure of the investigated 2D materials in this work (zigzag orientation): (a) graphene, (b) biphenylene, (c) borophene, (d) BC_3_, (e) h-BN, (f) C_3_N, (g–i) BC_6_N in 3 different structures (cyan atoms as carbon, pink as boron, blue as nitrogen).

Seven distinct two-dimensional material systems—BC_3_, BN, graphene, biphenylene, borophene, C_3_N, and BC_6_N—were constructed in both zigzag and armchair orientations utilizing the Material Studio,^40^ with each structure containing approximately 5000 atoms to establish well-defined initial configurations for subsequent MD simulations. For each material, the optimized primitive unit cell was first generated, then systematically replicated and oriented to produce supercells with either zigzag or armchair edge directions. All atomic types, bonding environments, and lattice vectors were meticulously validated to ensure consistency across the two orientations for each material. In this study, all materials were modeled using their most stable and widely accepted 2D allotropes. This approach ensures that the comparison of thermal-shock behavior accurately reflects realistic and experimentally relevant atomic structures. The canonical monolayer configurations for graphene, C_3_N, BC_3_, biphenylene, borophene, and h-BN were selected because they are recognised as the ground-state structures in the literature.^19,41–44^ These phases are well established and exhibit characteristic bonding topologies that influence their thermomechanical response. The only exception is BC_6_N, which has multiple energetically competitive arrangements due to different placements of boron (B) and nitrogen (N) within the hexagonal carbon lattice. To address this structural diversity, three distinct isomers of BC_6_N—*ortho*, *meta*, and *para*—were included in the study. These configurations represent all chemically meaningful placements of B and N atoms in a C_6_ ring and encompass a wide range of possible local electronic environments and bonding anisotropies.^45,46^ Details of each structure are available in Table S3, including simulation cell size and number of particles. Fig. 2 illustrates a portion of the simulation cell as the initial structure in the zigzag direction. While the armchair structures shown in Fig. 2S.

To investigate the thermal-shock behavior of these two-dimensional (2D) materials, molecular dynamics (MD) simulations were performed using the LAMMPS (Large-scale Atomic/Molecular Massively Parallel Simulator).^47^ Each structure was modelled with periodic boundary conditions in the in-plane (*x* and *y*) directions, while fixed (non-periodic) boundaries were applied in the out-of-plane (*z*) direction to mimic realistic constraints.^37^ The simulation system was subjected to energy minimization with box relaxation in the *x* and *y* directions to eliminate initial stress artifacts. The system was first relaxed in the NVT ensemble at a low temperature (0.1 K) to ensure that the initial configuration was well-defined and stable. This temperature choice was intended to avoid introducing significant initial thermal fluctuations that could interfere with the subsequent thermal shock process. The NVT relaxation was performed without a preceding NPT relaxation, as the focus of this simulation was on studying the material's response to localized thermal perturbations, rather than on achieving a particular pressure or density.

To induce thermal shock, a 10 Å slab at one end of the 2D sheet was the “hot” region, while a similar slab at the opposite end was the “fixed” region. Fixed boundary conditions in the fixed region prevented rigid motion and simulated a supported edge.

**Fig. 3:**
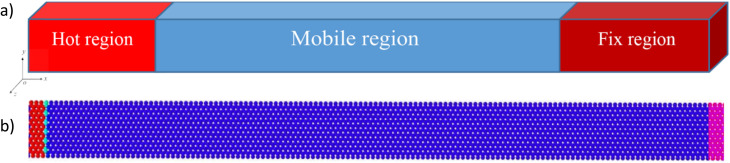
Illustrating (a) the schematic of defined regions and (b) simulation box.

After equilibrating, the hot region was suddenly heated to 1000 K *via* velocity rescaling, while the rest of the sheet remained at the low baseline temperature. This setup creates a sharply localized thermal gradient that induces a clear shock front and ensures that the heated zone spans several atomic rows to ensure numerical stability. After the thermal impulse, the system was switched to the NVE ensemble and propagated for 10 000 timesteps (timestep = 0.0005 fs), allowing the shock to evolve without thermostat interference. The choice of a tiny time step during relaxation and shock induction was necessary to ensure stable numerical integration, particularly at low temperatures where atomic vibrations can be highly sensitive to the time step. All simulations were performed for each zigzag and armchair orientation. The selected heating width and NVE duration were designed to capture the initial ballistic propagation of the shock while ensuring consistent results across different material configurations. The NVE ensemble (microcanonical ensemble) was employed for the thermal shock phase, allowing the system to evolve naturally without external energy exchange. The response of each material to thermal shock was analyzed in terms of temperature distribution, stress evolution, and atomic-level structural changes. The VMD^48^ and OVITO^49^ were used for post-processing visualization, while a custom MATLAB script was employed for plotting and related calculations. Wavefront positions were determined from smoothed stress profiles averaged over chunks, using a moving-average filter with a window size of 5. The leading stress peak was tracked without applying a specific amplitude threshold. Fig. 3a and b illustrate a schematic representation of the defined regions and an overview of the simulation box.

In molecular dynamics (MD) simulations of micro- and nanoscale heat transfer, evaluating stress distribution—whether local or global—is essential for analyzing the atomistic mechanical response of materials under thermal loading. Unlike continuum-based approaches, such as the finite element method (FEM), which relies on macroscopic stress–strain relationships, molecular dynamics (MD) calculates stress at the atomic scale based on statistical mechanics principles.^30,50,51^ In this work, atomic stress is computed using the virial formulation, which defines stress as an intensive quantity normalized over atomic volume. The virial stress tensor *σ*_*αβ*_, is given by eqn (1).^37^1
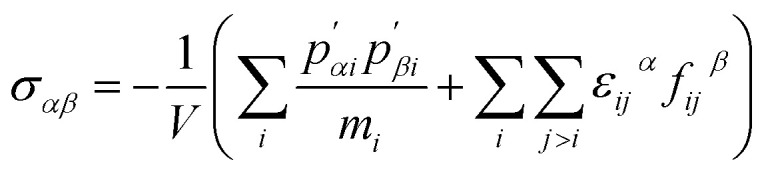
where *α*, *β* represent Cartesian components, *p*′ denotes the atomic momentum relative to the center of mass, *m*_*i*_ Is the mass of an atom *i*, *r*_*ij*_ Is the vector from the atom *i* to atom *j*, and *ij*. Is the force exerted by an atom *i* on atom *j*. The first term in the expression accounts for the kinetic contribution to stress. In contrast, the second term captures interatomic force contributions, which are particularly significant under non-equilibrium conditions, such as thermal shocks.

Atomic stress is a microscopic indicator of localized mechanical instability; elevated stress values indicate increased atomic mobility or a higher probability of bond rearrangement. This is crucial for understanding phenomena such as the propagation of stress waves and the initiation of fracture in two-dimensional materials. By averaging atomic stress over the entire system volume or specified regions, one can obtain macroscopic stress values that are identical to those predicted by continuum theories, thereby connecting atomistic insights to bulk mechanical behavior. While absolute stress-wave metrics may vary with boundary conditions and geometry, the relative trends reported here are intrinsic lattice structure and bonding characteristics.

To numerically analyze the decrease in stress-wave intensity due to thermal shock, we computed the decay rate of wave amplitude from MD simulation results. During thermal shock, a rapid temperature gradient generates a stress wave that propagates through the material lattice. As the wave moves away from the hot region, its amplitude decreases because of intrinsic energy dissipation mechanisms, including phonon–phonon interactions, lattice anharmonicity, and scattering from defects or boundaries. The decay rate is a critical parameter for characterizing this attenuation, providing insight into the material's ability to localize and dissipate thermal stress. The decay rate was assessed by analyzing the time-dependent evolution of atomic stress and displacement fields in the direction of wave propagation. For each time frame, the spatial distribution of a chosen stress component (*e.g.*, *σ*_*xx*_) was extracted, and the peak amplitudes were determined at progressively greater distances from the thermal shock front. The peak values were subsequently fitted using an exponential decay model, as shown in eqn (2).

2 *A*(*x*) = *A*_0_e^−*ax*^

where, *A*(*x*) is the wave amplitude at position *x*, *A*_0_ is the initial amplitude at the source, and *α* is the spatial decay rate. The decay rate *α* represents the spatial attenuation constant of the peak stress-wave amplitude caused by thermal shock. After shock initiation, the maximum longitudinal stress component (*σ*_{*xx*}_) was measured at successive points along the propagation path. The data fitting focused on the ballistic regime, from the edge of the heated region to just before boundary reflections or secondary waves interfere. This segment captures the initial, steady decay of stress amplitude, excluding areas affected by fixed boundaries or wave superposition. The decay constant was derived through nonlinear least-squares regression, with fit quality exceeding 0.95 in all cases, confirming that *α* reliably reflects intrinsic damping rather than numerical or size-related effects.^52^ Fig. S1 shows the fitting of the stress peaks of zigzag graphene to eqn (2).

The stress wave is the main ballistic mechanical response to the thermal impulse, whereas the temperature perturbation indicates the secondary, diffusive redistribution of energy after the stress wave passes.

In this study, material-specific empirical potentials were employed to accurately capture atomic interactions in each 2D system.^33,53^ The Tersoff potential was used for graphene, BN, BC_3_, C_3_N, BC_6_N, and biphenylene, given its established reliability in reproducing structural, mechanical, and thermal properties of covalently bonded 2D materials. For borophene, the Stillinger–Weber potential was chosen for its parameterization of the unique bonding environment of boron atoms in 2D sheets. All potentials have been validated in previous studies against experimental or first-principles data for lattice constants, cohesive energies, and elastic properties.^54–56^ Notably, the analysis focuses on comparative trends in thermal shock response rather than absolute property values, and uses normalized metrics to ensure meaningful comparisons across materials. Therefore, despite using different potentials, the simulation results provide consistent and robust insights into the relative thermal shock behavior of the seven 2D materials.

In this study, a “shock wave” is defined as a high-amplitude, nonlinear stress wave produced by an ultrafast, localized thermal impulse. It has a sharp leading edge, a significant reduction in amplitude, and differs from small-amplitude linear waves. The thermal shock strength is quantified by the immediate temperature increase Δ*T* in a specific region and the size of the heated area. The term “shock wave” is used here to denote the stress signal generated by an impulsive, localized thermal excitation. When a narrow slab experiences a rapid temperature increase, it induces a step-like rise in local stress, leading to a sharp stress-temperature front that propagates ballistically through the lattice. During the early propagation phase, this front retains a well-defined shape and moves with nearly constant velocity, much faster than thermal diffusion. The peak stress decreases steadily with distance from the heat source, which can be modeled exponentially. These characteristics distinguish this signal from typical dispersive stress waves induced by harmonic or stochastic excitation, supporting its classification as a thermal shock wave in this context.

The Tersoff potential is a bond-order potential that accounts for the influence of the local atomic environment on bond strength, making it particularly suitable for materials with directionally dependent covalent bonding. The total potential energy *E* in the Tersoff formulation is expressed in eqn (3).^57^3
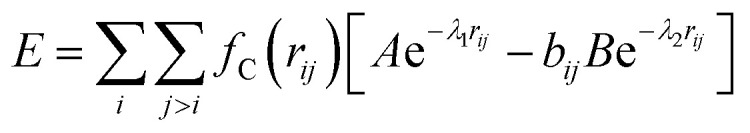
where *f*_C_(*r*_*ij*_) is a cutoff function, *r*_*ij*_ is the interatomic distance between atoms *i* and *j*, and *b*_*ij*_ The bond-order term incorporates many-body interactions and angular dependencies.

The Stillinger–Weber (SW) potential was used for borophene because it well describes the material's characteristic multi-center, flexible bonding structure. The SW potential includes both two-body and three-body interaction terms, making it more effective for materials such as borophene that do not conform rigidly to basic covalent bonding patterns. The total potential energy in the SW potential is defined by eqn (4).^58^4
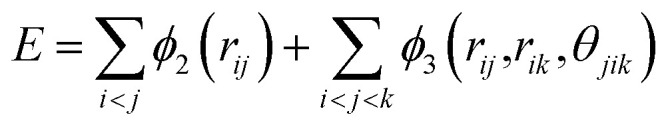
where *ϕ*_2_ is the two-body interaction and *ϕ*_3_ accounts for angular-dependent three-body interactions. The detailed potential parameters are presented in the SI, Tables S1 and S2.

To verify that the choice of simulation cell length does not affect our conclusions, we performed a length-convergence study on graphene. For each orientation, we compared two box lengths (1.0× and 2.0× the base cell) and measured the shock-front speed by tracking when the primary stress peak arrived at multiple probe positions. The longitudinal stress wave for each size is shown in Fig. S3. The measured propagation speeds differed by less than 0.5% across lengths, and the peak stress amplitude varied by less than 2%. Additionally, the first reflected pulse returned at a time that, in all cases, falls outside the NVE observation window used for primary analysis. Energy conservation in the NVE runs showed similar numerical drift across lengths. Overall, these results suggest that finite-cell length effects on wave speeds and orientation trends are negligible; thus, the results obtained with a 1× cell are representative of the intrinsic propagation behaviour documented in this work.

The uncertainty in the extracted wave speed and decay rate is constrained by the deterministic nature of the molecular dynamics framework employed. Stress-wave velocities were calculated through linear regression of shock-front position over time, with correlation coefficients above 0.99 in all cases, indicating very little fitting uncertainty. The spatial decay rate was determined by nonlinear least-squares fitting of peak stress amplitudes to an exponential decay model, yielding *R*^2^ values greater than 0.95. These fitting metrics give implicit confidence bounds for the reported values. Additionally, the variations observed across materials and propagation directions are substantially larger than the residual fitting scatter, confirming the robustness of the comparative results.

## 3 Results and discussions

The simulation results indicate that the material type influences the thermal shock response and exhibits directional anisotropy. Differences in decay rate, displacement, and stress-wave speed between armchair and zigzag orientations underscore how the atomic arrangement significantly influences dynamic behavior. Stress wave speed reflects a material's capability to transmit mechanical energy. Two metrics were assessed: *XY* Stress Wave Speed, indicating shear- or mixed-mode propagation, and *X* Stress Wave Speed, which primarily reflects the longitudinal component along the *x*-axis.

Following the localized thermal impulse, two distinct yet coupled transport processes begin: the propagation of temperature perturbations and the propagation of stress waves. The temperature change results from the redistribution of thermal energy through phonon-mediated heat transfer and occurs over a diffusive timescale. Conversely, the stress wave is generated by the immediate thermoelastic expansion induced by the temperature increase and propagates ballistically as a mechanical disturbance. In this study, stress-wave propagation is examined by monitoring the evolving longitudinal stress and atomic displacement fields in space and time, thereby enabling the extraction of wave speed and decay rate. Meanwhile, temperature changes are analyzed separately using spatially resolved kinetic temperature profiles to demonstrate thermal diffusion. The much faster propagation speed of the stress wave compared to the temperature perturbation clearly shows that these processes are distinguishable within the simulation timeframe, yet remain physically connected through thermoelastic effects.

**Fig. 4:**
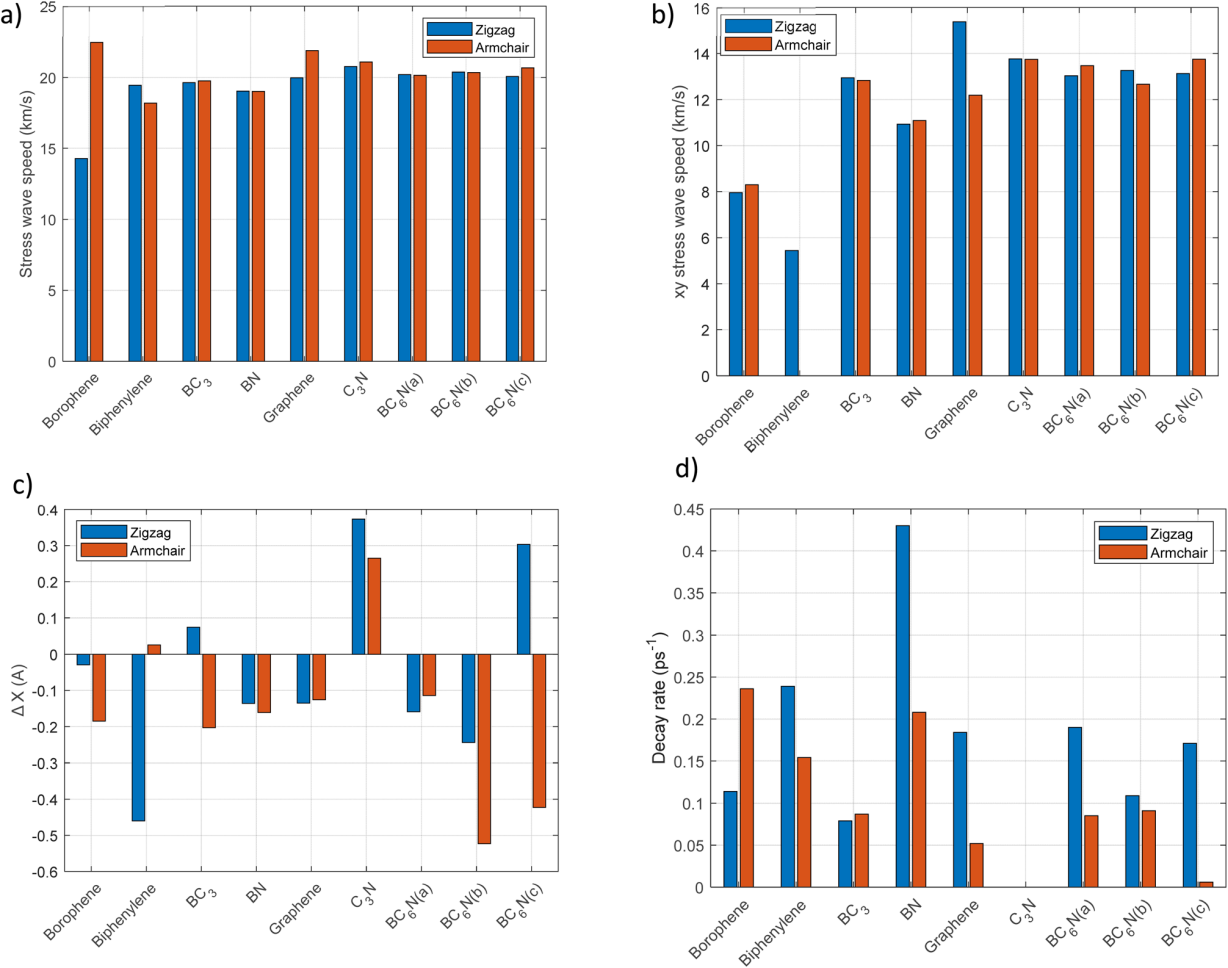
Comparing the results for each 2D material in zigzag and armchair directions. (a) Stress wave speed in km s^−1^ in the *X* direction. (b) Stress wave speed in km s^−1^ over the *xy* plane. (c) Atomic displacement, (d) measured decay rate in (ps^−1^).

Graphene and C_3_N demonstrate consistently high wave speeds in both directions. For example, C_3_N has a velocity of approximately 13.7 km s^−1^ in the *xy* plane and a total wave speed nearing 21 km s^−1^, a reflection of its strong covalent structure. Borophene shows notable anisotropy, with much faster wave propagation in the armchair direction (22.45 km s^−1^) compared to the zigzag direction (14.28 km s^−1^), even though it has a slightly higher *xy* speed when traveling in the zigzag direction. The BC_6_N exhibits moderate anisotropy, with longitudinal stress wave speeds averaging around 20 km s^−1^ and *xy* speeds at roughly 13 km s^−1^, indicating that atomic configuration is more influential than composition alone. Fig. 4a and b depict the bar plot of stress wave speed for each 2D material.

An initial comparison of stress wave propagation velocities reveals that, aside from borophene and graphene, most of the structures studied exhibit nearly identical wave speeds in both zigzag and armchair directions. Notably, borophene's propagation velocity is highly direction-dependent; specifically, it travels approximately 57% faster in the armchair direction than in the zigzag orientation. Likewise, in graphene, the armchair-propagation speed exceeds that in the perpendicular direction by roughly 10%. For the other materials, where the variation in propagation speed is under 6%, the wave velocities are deemed effectively equal. Additionally, the calculations indicate that the shear stress wave propagation speed (*S*_*xy*_) is largely unaffected by the structure's configuration or the direction of thermal shock application. The differences between the zigzag and armchair directions are minimal. This consistent pattern is seen across all two-dimensional materials studied, including borophene, despite its notable anisotropy in longitudinal wave propagation.

Fig. 4c shows that the Δ*x* values represent the net atomic displacement along the *x*-axis due to thermal shock. The sign of Δ*x* reveals the nature of the deformation: negative values, observed in borophene, BN, and graphene, imply contraction or possible out-of-plane buckling, leading to in-plane displacement. BC_3_ exhibits directional behavior: a positive Δ*x* (0.075 Å) in the armchair orientation and a negative Δ*x* (−0.203 Å) in the zigzag orientation. C_3_N is notable once more, showing positive displacements in both directions, with the armchair orientation exhibiting a larger value (0.373 Å) than the zigzag (0.265 Å), suggesting uniform expansion under thermal loading. These findings align with the displacement–length plots, in which the magnitude of the peak displacement and the waveform shape reflect structural deformation patterns and their propagation characteristics. A negative Δ*x* likely indicates compressive or buckling effects under thermal excitation, especially in materials with lower stiffness. Conversely, positive Δ*x* denotes thermal expansion or structural stretching, as seen in C_3_N.

Fig. 4d illustrates the decay rate values in (ps^−1^) for the analyzed 2D materials. Notable trends specific to each material include: borophene displays a substantially higher decay rate in the armchair direction (0.236 ps^−1^) than in the zigzag direction (0.114 ps^−1^), indicating more effective thermal energy dissipation along the armchair. In contrast, BN and biphenylene show the opposite trend, with greater decay in the zigzag orientation. Graphene also exhibits significant anisotropy, with decay rates of 0.052 ps^1^ (armchair) and 0.184 ps^1^ (zigzag). An exciting case is C_3_N, which shows a decay rate of 0 ps^−1^ in both directions, indicating an undamped thermal wave—a hallmark of exceptional thermal stability and likely reflected in the temperature–length plots as a consistent thermal profile over time. BC_6_N(*c*) displays minimal decay in the armchair (0.006 ps^−1^) but considerable decay in the zigzag (0.171 ps^−1^), further highlighting the significance of structural directionality. These results emphasize the impact of crystallographic orientation and bonding topology on thermal energy dissipation.

Decay rates reveal how quickly materials dissipate injected stress energy, ranging from nearly zero in the armchair direction for C_3_N to 0.236–0.239 ps^−1^ in borophene and biphenylene, indicating diverse dissipation mechanisms. High wave velocity does not always mean low damping: borophene has moderate speeds but high decay rates, while C_3_N propagates quickly with minimal damping. This shows that wave speed is dictated by harmonic stiffness, whereas damping arises from anharmonic scattering, mode mixing, and topological irregularities. Materials with non-hexagonal rings or heteroatomic substitutions experience larger bond-length and angle fluctuations, boosting anharmonic effects and phonon scattering. This explains the pronounced decay of biphenylene and BC_3_, in which structural distortions localize vibrations and cause energy leakage. Borophene's decay varies with direction (0.236 ps^−1^ in armchair *vs.* 0.114 ps^−1^ in zigzag), due to anisotropic coupling between longitudinal modes and flexible low-frequency branches. Quasi-1D ridges channel stress energy into out-of-plane modes, accelerating attenuation. The considerable transverse decay amplitudes in borophene and biphenylene suggest substantial transverse destabilization, a sign that topologically frustrated materials shift stress energy more readily into non-propagating directions. The BC_6_N isomers demonstrate how isomeric differences influence thermal-shock response: despite identical composition, *para*-, *meta*-, and *ortho*- configurations show distinct decay rates (*γ* = 0.00–0.190 ps^−1^) and directional behaviors, stemming from variations in bond polarity, mass distribution, and heteroatomic substitution, which affect phonon spectra and scattering channels. For example, BC_6_N(*c*) exhibits nearly zero damping along the armchair but strong damping along the zigzag, highlighting how small atomic changes can affect energy dissipation. Overall, this emphasizes that thermal-shock resistance in BCN-based materials depends heavily on isomerism—a novel insight from this work.

Atomic displacement governs stress wave propagation during thermal shock by reflecting the lattice's response to temperature gradients. Rapid heating induces localized atomic vibrations, thereby initiating a stress wave. Smaller, uniform displacements in materials such as graphene enable high-velocity, coherent wave propagation due to strong, symmetric bonds. Larger or irregular displacements, seen in biphenylene and borophene, disrupt coherence and cause faster decay. Variations in bond strength, coordination, and symmetry influence how mechanical disturbances are transmitted. Analyzing atomic displacement reveals insights into wave attenuation, anisotropy, and thermal shock resistance. Quantitative data indicate that materials such as graphene exhibit displacements below ∼0.4 Å, which correlate with high wave speeds and minimal loss. Larger displacements in BC_3_ and biphenylene lead to slower, more damped waves. Borophene's directional anisotropy affects wave speed and damping, with lower displacements along the armchair direction supporting faster propagation. Overall, the magnitude and smoothness of displacement determine the stress-wave speed, attenuation, and thermal-shock resistance.

The results indicate that there is no strict one-to-one correlation between wave speed and decay rate in the analyzed materials, as these phenomena originate from distinct lattice mechanisms. Wave speed depends on elastic stiffness and bond connectivity, which govern the propagation of mechanical disturbances. Conversely, the decay rate is affected by internal damping processes, such as phonon–phonon scattering, structural anharmonicity, variations in coordination, and local topological irregularities, which dissipate energy during wave propagation. This distinction elucidates the different behaviors observed in C_3_N and borophene: C_3_N exhibits a high wave speed with minimal decay, attributable to its symmetric, uniformly coordinated sp^2^ network, which mitigates anharmonic interactions and preserves wave coherence over extended distances. Borophene, particularly in the armchair orientation, exhibits a high propagation velocity but also undergoes significant decay. Its in-plane stiffness facilitates rapid transmission; however, its anisotropic properties, buckling phenomena, and bond heterogeneity promote damping and energy scattering, resulting in a more rapid attenuation of wave amplitude. These findings underscore that wave speed and decay are not inherently correlated; instead, decay is more strongly influenced by a material's anharmonicity and damping characteristics than by its propagation velocity. Such decoupling is critical for evaluating thermal shock resistance, as a material may efficiently transmit stress waves while dissipating energy swiftly or maintaining coherence despite a moderate wave speed.

Considering the anisotropic mechanical and thermal characteristics of the structures examined, it was expected that the wave propagation velocity would demonstrate dependence on direction. Consequently, simulations were performed for both zigzag and armchair configurations, and the results are summarized in Table 1.

**Table 1:** Mechanical properties for different configurations

Structure	Stress wave speed (km s^−1^)		*xy* stress wave speed (km s^−1^)		Δ*x* (Å)		Decay rate *α* (ps^−1^)	
	Zigzag	Armchair	Zigzag	Armchair	Zigzag	Armchair	Zigzag	Armchair
Borophene	14.277	22.446	8.297	7.955	−0.185	−0.029	**0.114**	0.236
Biphenylene	19.443	18.181	Not measurable	5.44	0.026	−0.460	**0.239**	0.154
BC_3_	19.634	19.757	12.838	12.955	−0.203	0.075	**0.079**	0.087
BN	19.030	19.010	11.090	10.934	−0.161	−0.136	**0.430**	0.208
Graphene	19.967	21.869	12.193	15.381	−0.126	−0.135	**0.184**	0.052
C_3_N	20.742	21.065	13.742	13.765	0.265	0.373	0.0	0.0
BC_6_N(*a*)	20.202	20.146	13.468	13.042	−0.114	−0.159	0.190	0.085
BC_6_N(*b*)	20.379	20.340	12.672	13.261	−0.523	−0.244	0.109	0.091
BC_6_N(*c*)	20.061	20.653	13.751	13.140	−0.423	0.303	0.171	0.0

In total, a thermal shock of 1000 K causes a displacement of a few tenths of an angstrom (less than 0.5 Å), leading to localized tensile or compressive deformation in the structure's initial region. This results in a peak in the stress profile, triggering the development of a forward-propagating wave.

**Fig. 5:**
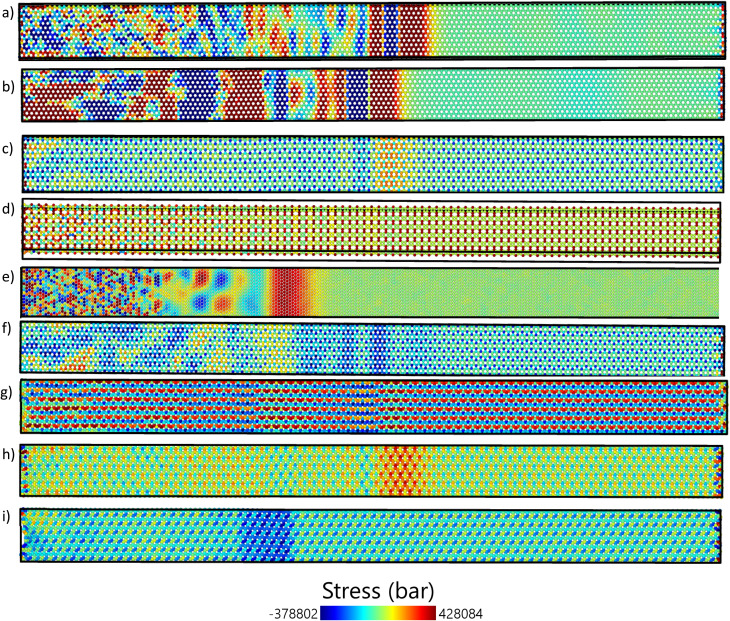
The thermal stress disturbance in the *X* direction of zigzag (a) graphene, (b) h-BN, (c) BC_3_, (d) biphenylene, (e) borophene, (f) C_3_N, (g) BC_6_N(1), (h) BC_6_N(2), (i) BC_6_N(3) at 1 ps.

In this study, a stress-wave quantity is deemed unmeasurable if no coherent, forward-propagating stress-wave peak can be identified and tracked in the stress profiles, despite variations in numerical parameters such as time step and repeated simulations with different initial velocities. Under these circumstances, extracting wave speed lacks physical meaning, so the quantity is reported as not measurable. On the other hand, decay rates marked as zero suggest the fitted attenuation constant is below the numerical resolution of the exponential fit within the examined propagation window. In such cases, the decay constant is statistically indistinguishable from zero within its confidence interval, indicating near-undamped propagation rather than complete absence of dissipation. As shown in Fig. 5S, biphenylene's *σ*_{*xy*}_ profiles display rapid decay and irregular fluctuations instead of a clear peak. To confirm this is not a numerical artifact, additional simulations were conducted with a tenfold smaller time step (from 0.5 fs to 0.05 fs). As shown in Fig. S6(b), no qualitative differences or detectable propagating shear-stress peaks are observed compared to the original case in Fig. S6(a). The simulation was also repeated using a different random initial-velocity distribution. As shown in Fig. S6(c), the stress evolution remains essentially the same, indicating that the absence of a measurable shear-stress wave is not due to initial condition noise. Similar rapid decay of the *σ*_{*xy*}_ signal was observed for biphenylene in the armchair orientation.

Materials with high wave speeds show rapid front propagation, while those with significant decay rates exhibit diminished wave amplitudes over time. According to eqn (2), an increased damping rate leads to more significant wave attenuation, while a decreased damping rate results in less wave attenuation. Among the structures analyzed, the wave amplitude in biphenylene decreases more quickly than in the other configurations, as shown in the table, indicating the highest damping rate. In contrast, borophene experiences a much slower decline in stress peak amplitude, and its damping rate is lower than that of the other structures. For the biphenylene structure, measuring the propagation speed of the shear stress wave (*S*_*xy*_) proved impossible, as no clear forward-moving peak is visible in the corresponding graph (Fig. 4). This absence may be due to either the high damping rate or the structure's unique geometry, which neutralizes shear stress.

To quantitatively rationalize stress-wave damping and coherence, we correlate the extracted spatial decay rate *α* with lattice anharmonicity, mass distribution, and structural topology. Materials with highly coherent propagation, such as graphene and C_3_N, exhibit negligible attenuation, with decay rates approaching zero (*α* ≈ 0–0.05 ps^−1^) and high stress-wave velocities of ∼20–21 km s^−1^, indicating weak phonon–phonon scattering and near-harmonic lattice response. In contrast, biphenylene and BC_3_ display substantially higher decay rates (*α* ≈ 0.15–0.24 ps^−1^ in the armchair direction), reflecting enhanced phonon scattering caused by non-hexagonal ring networks and heterogeneous bonding environments. Borophene shows strong anisotropy, with *α* ≈ 0.24 ps^−1^ along the armchair direction compared to ≈0.11 ps^−1^ along zigzag, accompanied by larger out-of-plane atomic displacements (up to ∼0.37 Å). This behavior quantitatively confirms that intrinsic buckling and directional bonding amplify anharmonic dissipation and suppress wave coherence. Collectively, these metrics demonstrate that thermal-shock damping in 2D materials is governed not only by chemical composition but also by lattice symmetry, coordination, and mass distribution, providing a quantitative framework for comparing transient thermomechanical resilience.

Fig. 5 illustrates the thermal stress distribution in the zigzag orientation along the *x*-direction at *t* = 1 ps, providing a visual representation of stress propagation. High—quality complete simulation trajectories are provided in SI Movies. The extent of stress wave propagation differs by material; for example, C_3_N (Fig. 5f) exhibits a particularly sharp and well-defined wavefront, indicating efficient and less dispersive wave propagation, while graphene (Fig. 5a) also displays a relatively straight forward wavefront. Borophene (Fig. 5e) features a distinct high-stress area (marked in red) near its wavefront. In contrast, materials like h-BN (Fig. 5b) and biphenylene (Fig. 5d) exhibit more complex, diffuse stress patterns, which may suggest greater scattering or more intricate wave interactions within these structures in the zigzag orientation.

Each material exhibits a unique stress profile, reflecting the differences in its atomic structure and bonding attributes. For instance, graphene, known for its robust sp^2^ carbon lattice, likely exhibits a different stress response than borophene, which has a more complex structure. The BC_6_N variants (1, 2, and 3) are expected to exhibit varied stress responses due to structural differences that affect their mechanical properties under thermal shock. Graphene and C_3_N show relatively uniform stress wave propagation with distinct wavefronts in both directions. This is evident in their high-stress wave speeds (20–21 km s^−1^) and low decay rates, especially for C_3_N, which exhibits a decay rate of 0 ps^−1^. The stress remains sharp and continuous along the sheet's length, making them ideal for thermal-shock applications where minimal energy dissipation is essential. The h-BN behaves similarly to graphene, albeit with slightly greater stress diffusion, which aligns with its moderately high wave speeds (∼19 km s^−1^) and a higher decay rate in the zigzag direction (0.184 ps^−1^) than in the armchair direction (0.052 ps^−1^).

**Fig. 6:**
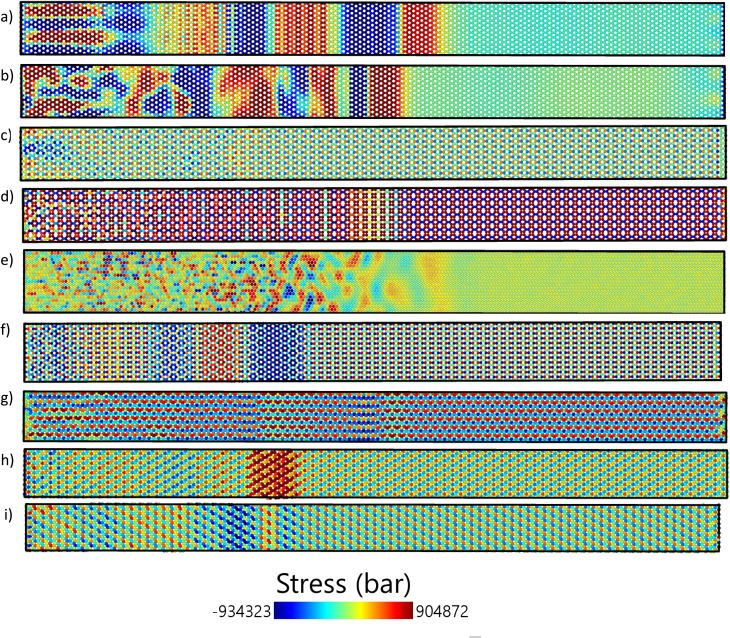
The thermal stress distribution in the *X* direction of armchair (a) graphene, (b) h-BN, (c) BC_3_, (d) biphenylene, (e) borophene, (f) C_3_N, (g) BC_6_N(1), (h) BC_6_N(2), (i) BC_6_N(3) at 1 ps.

Fig. 6, like Fig. 5, emphasizes the thermal-stress disturbance in the *X* direction for the armchair configuration of the same 2D materials at *t* = 1 ps. The analyzed materials are the same as those in Fig. 5: graphene, h-BN, BC_3_, biphenylene, borophene, C_3_N, and BC_6_N (1, 2, and 3). The stress profiles in the armchair direction may differ from those in the zigzag direction due to variations in edge geometries and bonding arrangements. For instance, graphene's armchair edges could result in a different stress propagation pattern compared to its zigzag edges, possibly influencing the material's thermal shock response. Likewise, the BC_6_N variants may exhibit distinct stress behaviors in the armchair configuration, influenced by their compositional differences. A comparison between Fig. 5 and 6 reveals the variations in stress disturbances between zigzag and armchair configurations, underscoring the anisotropic characteristics of thermal shock responses in these 2D materials.

Building upon the zigzag stress distributions depicted in Fig. 5 and 6 displays the thermal stress distribution in the *x*-direction for the armchair orientation at *t* = 1 ps. Consistent with the zigzag cases, C_3_N (Fig. 6f) exhibits a distinct and sharp wavefront in the armchair direction. Graphene, in the armchair direction (Fig. 6a), reveals an initial compressive (blue) region followed by a tensile (red) region. A visual comparison between Fig. 5 and 6 for the same materials often reveals notable anisotropic stress propagation patterns, in which differences in shape, clarity, and internal structure of the wavefront can vary significantly with crystallographic orientation. For example, the stress patterns in h-BN (Fig. 5b*vs.*6b) and biphenylene (Fig. 5d*vs.*6d) demonstrate marked differences between the two orientations, underscoring the directional dependence of the material's thermal shock response. Borophene (Fig. 5e and 6e) also displays variations in the stress distribution pattern, although high-stress regions are noticeable in both orientations.

Borophene exhibits significant anisotropy. In the zigzag direction (Fig. 5e), the stress profile is more spread out, whereas in the armchair direction (Fig. 6e), a sharper and quicker wavefront is observed. This is associated with the greater stress wave speed (22.45 km s^−1^ in armchair compared to 14.28 km s^−1^ in zigzag) and decay rate (0.236 ps^−1^ in armchair *versus* 0.114 ps^−1^ in zigzag) shown in the table of results.

BC_3_ and biphenylene exhibit reduced and fragmented stress distributions, particularly along the zigzag direction. Biphenylene, for instance, exhibits a nearly nonexistent *xy* stress wave speed in the zigzag configuration, which suggests suppressed or unclear shear wave propagation. These results are further illustrated by the visual distribution and attenuation of stress in Fig. 5d and 6d.

BC_6_N structures (Fig. 5g–i and 6 g–i) show variability in their orientations. While BC_6_N(1) and BC_6_N(3) present relatively consistent stress profiles, BC_6_N(2) appears more scattered in the zigzag direction, corresponding to its higher decay rate and anisotropic displacement values. These variations highlight the subtle structural differences among the 2D materials.

The origins of thermal-shock resistance in these 2D materials are supported by links between their lattice features and shock-response metrics. Bond strength and coordination affect the stress-wave velocity and decay: materials with uniform sp^2^ networks and stiff bonds, like graphene and C_3_N, support fast wave transmission (∼20–21 km s^−1^) with little attenuation (*α* ≈ 0–0.05 ps^−1^), indicating weak anharmonic scattering and efficient energy transfer. Conversely, lattices with complex topologies and non-hexagonal rings, such as biphenylene and BC_3_, show slower wave speeds and higher decay rates (*α* up to ∼0.24 ps^−1^), reflecting increased phonon scattering due to bond heterogeneity. Anisotropic effects are evident in borophene, where armchair and zigzag directions differ in decay rates, speeds, and out-of-plane displacements, aligning with its buckled and direction-dependent bonding. These trends demonstrate that bonding topology, uniformity, and lattice anisotropy influence thermal-shock damping and coherence, providing a quantitative basis for these mechanisms.

The SI Movie contains all thermal stress disturbance simulations for both zigzag and armchair orientations, as well as for *x*-stress and *xy*-stress. For more details, please refer to the SI file.

**Fig. 7:**
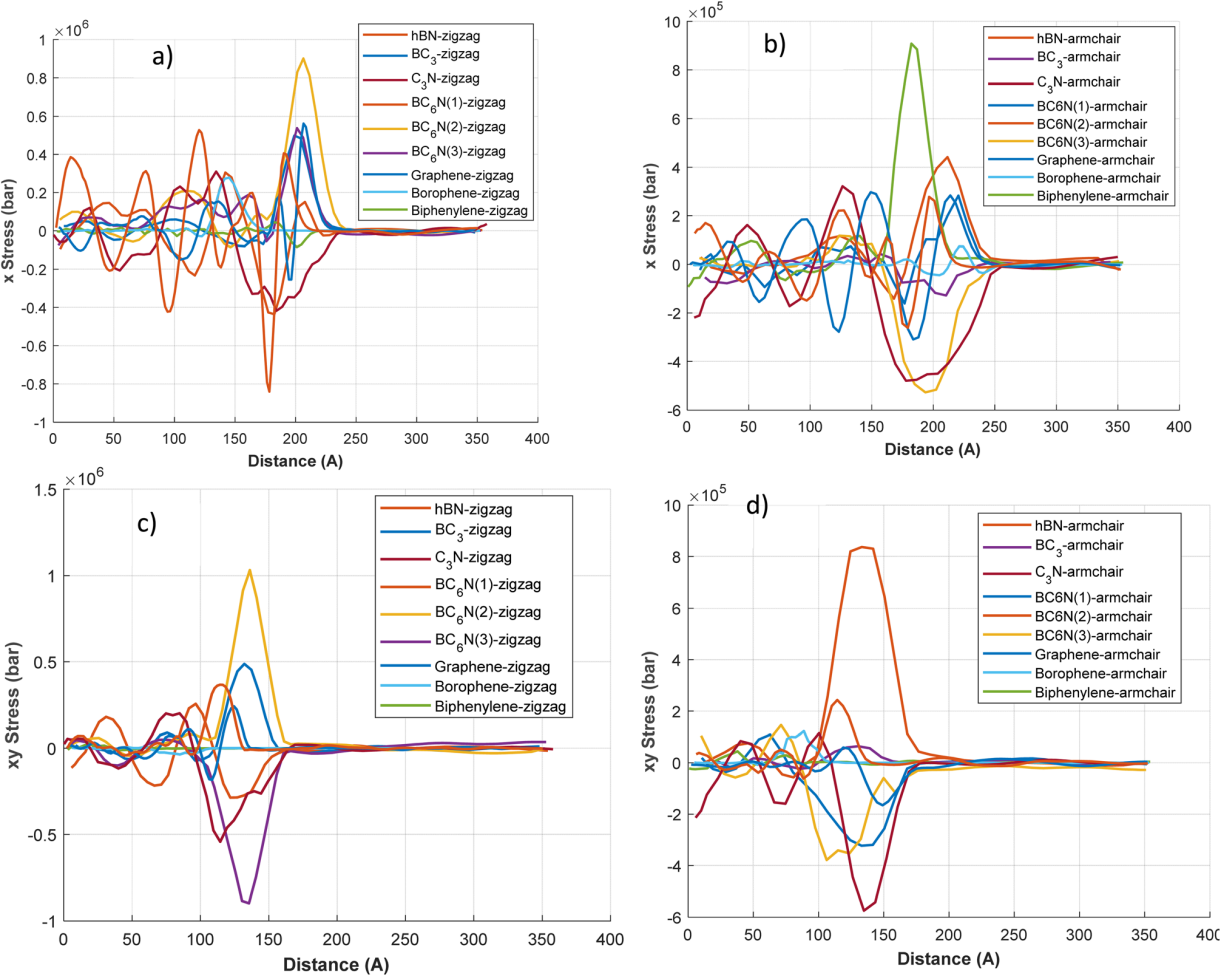
The longitude and shear stress wave along the length of the 2d materials in different directions at *t* = 1 ps. (a) longitude stress (*x* stress) for zigzag direction, (b) longitude stress (*x* stress) for armchair direction, (c) shear stress (*xy* stress) for zigzag direction, (d) shear stress (*xy* stress) for armchair direction.

Fig. 7 illustrates the stress plots for the 2D materials, although the excerpt lacks specific details about the coordination or direction. These plots likely enhance the stress disturbances depicted in Fig. 5 and 6, offering additional insights such as stress evolution over time and stress distribution in other axes (*e.g.*, *Y* or *Z*). The stress patterns in these plots may indicate how thermal shock triggers mechanical responses, leading to tensile or compressive stresses throughout the material. For example, materials such as graphene and h-BN, known for their high mechanical strength, could exhibit more uniform stress distributions. At the same time, borophene (BC_3_) may exhibit higher stress concentrations due to its distinctive structural features. Understanding these plots is crucial for evaluating the mechanical stability of materials under thermal shock.

Fig. 7 illustrates the variations in *X* stress (longitudinal) and *XY* stress (shear or biaxial component) with distance, for zigzag and armchair orientations. Regarding *X* stress, materials exhibit characteristic wave profiles, typically displaying leading compressive (negative stress) regions followed by tensile (positive stress) regions, or *vice versa*. In the zigzag direction (Fig. 7a), graphene-zigzag exhibits a significant tensile peak surpassing 0.8 × 106 bar around 175 Å, preceded by a region of compressive stress. Borophene-zigzag exhibits a sizable tensile peak of approximately 0.5 × 10^6^ bar at a distance of about 130 Å. C_3_N-zigzag features a sharp, narrower tensile peak, while biphenylene-zigzag displays notable oscillations with several high-magnitude peaks. In the armchair direction (Fig. 7b), the graphene armchair exhibits a substantial tensile peak of approximately 9 × 10^5^ bar (refer to the 10^5^ scale) at around 150 Å. The borophene armchair also exhibits a significant tensile peak, and the biphenylene armchair shows the highest tensile peak in this category, at around 150 Å. The *XY* Stress components are depicted in Fig. 7 (bottom panels). For the zigzag direction (Fig. 7c), biphenylene-zigzag exhibits exceptionally high *XY* stress, with peaks of approximately 1 × 106 bar (tensile) and −0.8 × 106 bar (compressive). Borophene-zigzag also indicates considerable *XY* stress peaks, while graphene-zigzag shows a notable *XY* stress peak near 150 Å. In the armchair direction (Fig. 7d), *XY* stresses are generally an order of magnitude lower, aligning with the axis scaling. Nevertheless, the biphenylene-armchair still presents the most significant *XY* stress peaks in this segment, reaching approximately 8 × 10^5^ bar. The variations in stress magnitudes and the distinct profiles of tensile and compressive regions between the orientations underscore considerable stress anisotropy.

These plots confirm the direction of stress propagation. Materials such as graphene and C_3_N exhibit elongated wavefronts aligned with the propagation axis, indicating uniaxial stress transport. Conversely, borophene and BC_3_ exhibit scattered contours, particularly in the zigzag direction, highlighting their greater anisotropy and decay. In biphenylene, stress is more localized and fragmented, likely due to its complex bonding, which inhibits long-range stress transport in specific directions. The SI, Movie S1, displays the evolution of longitudinal and shear stresses over distance during the simulation, for zigzag and armchair orientations.

**Fig. 8:**
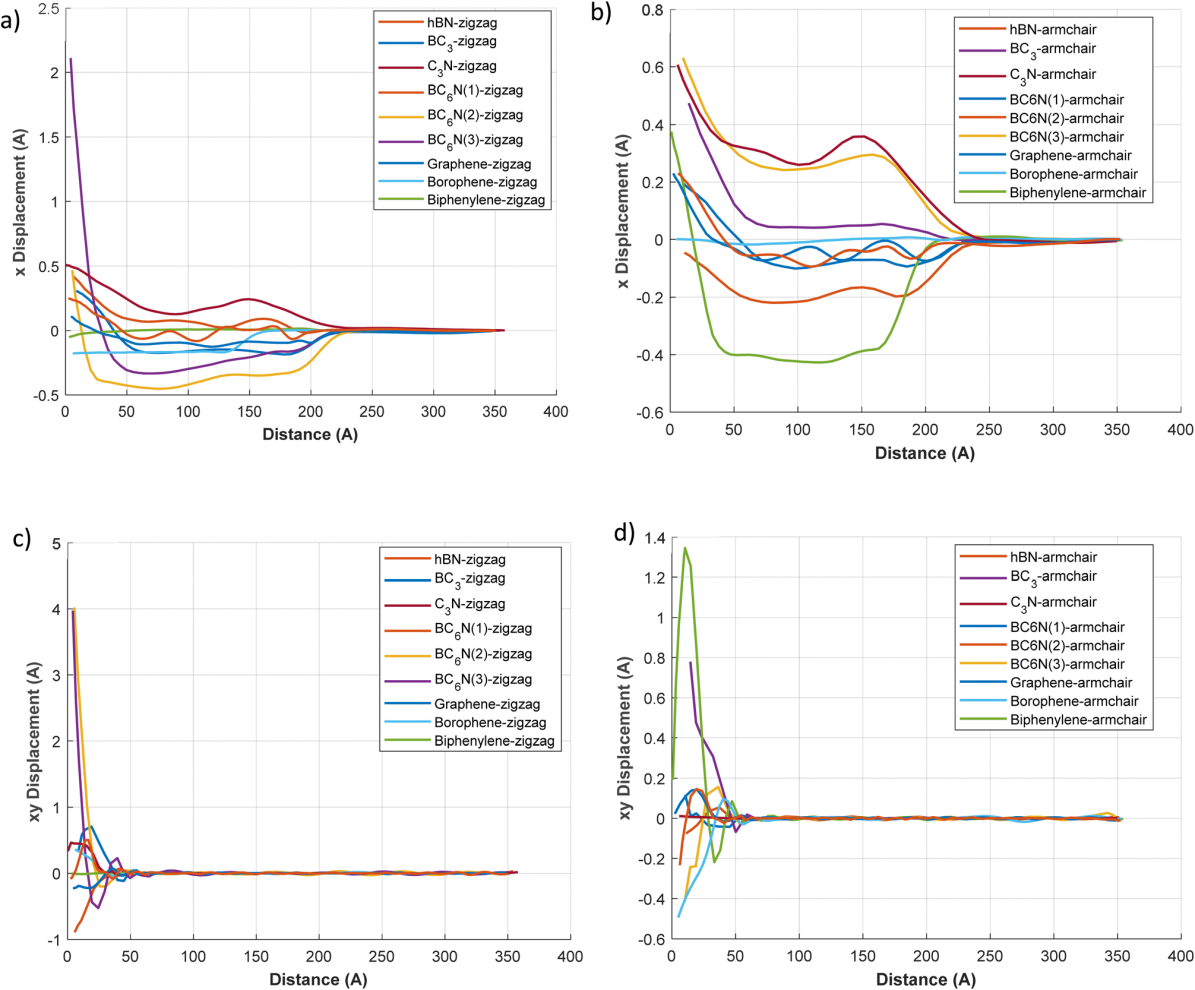
The longitude and shear displacement along the length of the 2d materials in different directions at *t* = 1 ps. (a) longitude displacement (*x* displacement) for zigzag direction, (b) longitude displacement (*x* displacement) for armchair direction, (c) shear displacement (*xy* displacement) for zigzag direction, (d) displacement stress (*xy* displacement) for armchair direction.

Fig. 8 depicts the displacement plots of 2D materials, highlighting the atomic or lattice displacements caused by thermal shock. These plots likely illustrate the degree of structural deformation or vibrational amplitudes resulting from the thermal disturbance. Materials such as graphene, known for its rigid lattice, may exhibit minimal displacements, whereas borophene, with its flexible, anisotropic structure, may show more significant displacements. The displacement behavior of BC_6_N variants is expected to vary depending on their specific atomic configurations, potentially indicating intermediate responses between graphene and borophene. Understanding these displacement profiles is crucial for evaluating the structural integrity and dynamic response of these materials to rapid thermal fluctuations.

In the zigzag direction for *X* displacement (Fig. 8a), most materials, including graphene-zigzag and hBN-zigzag, show negative displacement (contraction) in the wave region, with graphene-zigzag reaching a peak of about −0.2 Å. Borophene-zigzag starts with a significant positive *X* displacement of approximately 2.3 Å at *x* = 0, which quickly shifts to negative. C_3_N-zigzag presents a small positive displacement. In the armchair direction (Fig. 8b), C_3_N-armchair (∼0.65 Å) and BC_3_-armchair (∼0.3 Å) exhibit positive *X* displacement (expansion). The graphene armchair exhibits a marked negative *X* displacement, peaking at around −0.5 Å, whereas the Borophene armchair initially displays a positive displacement that later becomes negative. These displacement profiles align with previously reported Δ*x* values.

The *XY* displacement, which shows motion perpendicular to the central wave-propagation axis within the plane, is illustrated in Fig. 8c and d. In the zigzag direction (Fig. 8c), biphenylene-zigzag reveals a considerable initial *XY* displacement, peaking at about 4.5 Å near *x* = 0. Borophene-zigzag also indicates a substantial initial *XY* displacement of roughly 1.5 Å. Other materials typically portray smaller *XY* displacements in this orientation. For the armchair direction (Fig. 8d), the biphenylene armchair again demonstrates the highest initial *XY* displacement, reaching a peak of approximately 1.3 Å near *x* = 0. The borophene armchair also displays a significant initial *XY* displacement. Clear anisotropic displacement behavior is observed when comparing zigzag and armchair directions; for instance, borophene's initial *X* displacement is significantly larger in the zigzag orientation compared to the armchair, and biphenylene shows considerable *XY* displacement in both orientations, although the magnitudes differ.

C_3_N shows positive displacement in both orientations, with a greater magnitude in the armchair direction. This indicates thermally induced expansion and corresponds with previously measured Δ*x* values (0.373 Å for armchair, 0.265 Å for zigzag), further supporting the absence of thermal damping. Graphene and BN exhibit negative displacement in both directions, consistent with slight thermal contraction or buckling during rapid heating. Materials like BC_3_ and borophene show direction-dependent displacement; BC_3_ notably expands in the armchair direction while contracting in the zigzag direction, corresponding with the displacement values (0.075 Å for armchair, −0.203 Å for zigzag direction). BC_6_N samples exhibit complex displacement patterns, displaying both positive and negative values depending on the variant and orientation, which highlights their structural flexibility and response to thermal loading. The movie S1 displays all simulation stress-distance and displacement–distance plot animations for both zigzag and armchair orientations, as well as for the longitudinal (*x*) and shear (*xy*) stresses. Refer to the SI file for additional details.

**Fig. 9:**
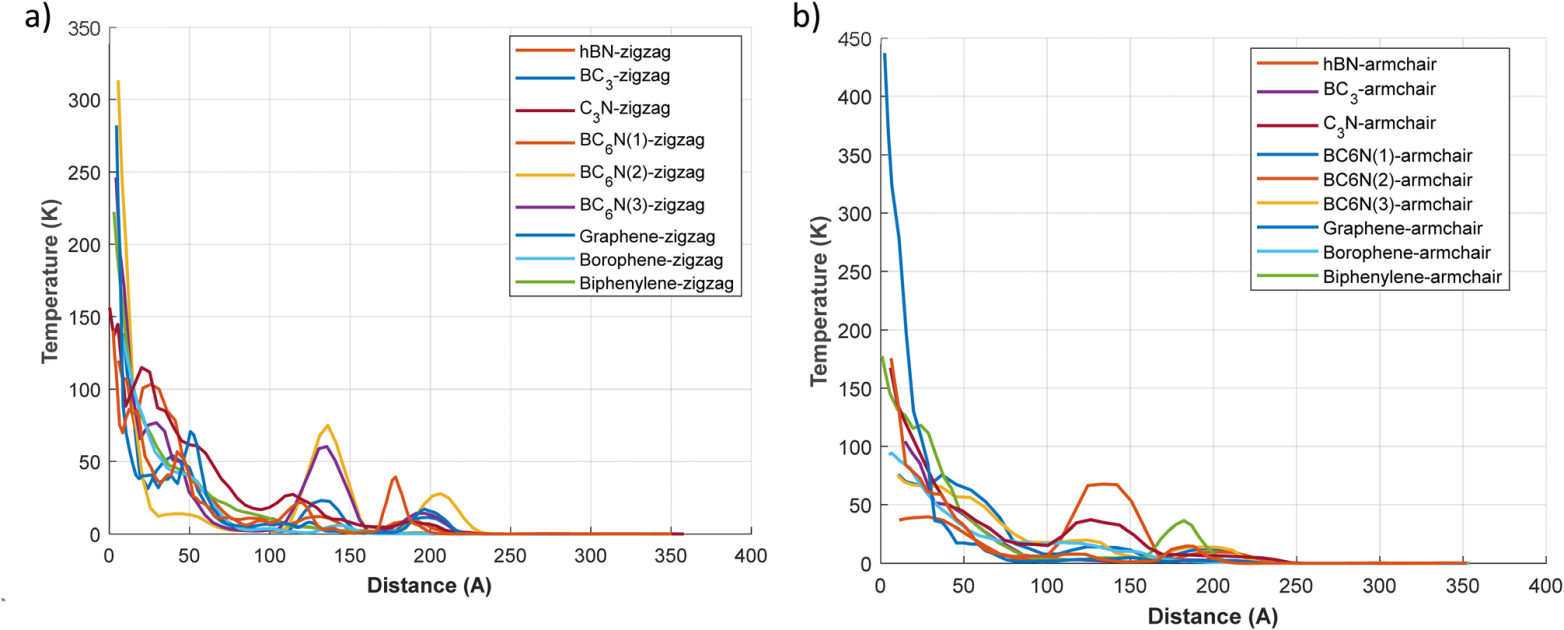
The temperature of 2D materials over the length of 2d materials at *t* = 1 ps. (a) Temperature–distance plot for zigzag direction, (b) temperature–distance plot for armchair direction.

Fig. 9 illustrates the temperature distribution across the 2D materials by analyzing profiles along the zigzag (Fig. 9a) and armchair (Fig. 9b) crystallographic orientations under thermal-shock conditions. Generally, most materials show a significant temperature drop near the origin (*x* = 0), followed by a more gradual decline, reflecting the dissipation of thermal energy during propagation. A notable exception is C_3_N, which, in both configurations, exhibits an unusually sharp initial temperature drop within about the first 25 Å. Following this, the temperature in C_3_N remains very low and decays extremely slowly, enabling the thermal disturbance to extend much further than in other materials, consistent with a nearly zero thermal decay rate. For both C_3_N and graphene, the temperature profiles remain relatively flat along the material length, indicating minimal decay and efficient thermal wave conduction, consistent with their known high thermal conductivities.

Thermal-shock-induced stress wave decay is closely related to bond oscillations and how mechanical energy redistributes into lattice vibrations. Materials like graphene and C_3_N, with strong, uniform bonds, show minimal bond-length oscillations under thermal shock. This results in nearly undamped wave propagation with decay rates around zero (*α* ≈ 0–0.05 ps^−1^) and high wave speeds of approximately 20–21 km s^−1^. On the other hand, structures with heterogeneous bonds and distorted ring configurations, such as biphenylene and BC_3_, display significant bond oscillations, indicated by larger atomic displacements and higher decay rates (*α* ≈ 0.15–0.24 ps^−1^). These oscillations enhance anharmonic phonon interactions, speeding up the loss of coherent stress-wave energy into localized vibrations. Borophene exemplifies this through its strong directional properties: the armchair direction shows larger out-of-plane bond oscillations (up to ∼0.37 Å) and a higher decay rate (*α* ≈ 0.24 ps^−1^) compared to the zigzag (*α* ≈ 0.11 ps^−1^). This illustrates how bond oscillation and lattice buckling together amplify energy dissipation. Overall, these trends highlight bond oscillation amplitude and directional stiffness as key factors influencing shock-wave decay and thermal energy dissipation in two-dimensional materials.

The comparison of zigzag and armchair orientations demonstrates anisotropic thermal transport. For example, graphene in the armchair direction maintains a slightly higher temperature along its length than in the zigzag direction. This anisotropic characteristic likely reflects the lattice's inherent properties, which may generate localized hotspots or temperature gradients depending on the material's thermal conductivity and structural integrity. Varying edge geometries in armchair and zigzag configurations affect phonon transport and heat dissipation.

In contrast to materials with high thermal stability, borophene, biphenylene, and BC_3_ display sharper temperature gradients, especially pronounced in the zigzag direction for both borophene and BC_3_. These gradients indicate elevated decay rates, suggesting significant energy loss from lattice damping and phonon scattering. As a result, materials like borophene and biphenylene exhibit a relatively rapid temperature decline in both orientations, reaching baseline temperatures within 150–200 Å.

Additionally, certain materials showcase more intricate temperature profiles. For instance, hBN-zigzag, BC_3_-zigzag, and, particularly, hBN-armchair exhibit secondary peaks or shoulders in their temperature distributions. Specifically, the hBN-armchair shows a prominent secondary temperature peak at around 150 Å, which may indicate reflections or energy conversions within the material. The BC_6_N variants exhibit various temperature-length behaviors, reflecting their structural polymorphism; some configurations show a consistent decrease in temperature, while others indicate partial energy retention, aligning with their intermediate decay rates and stress wave velocities. These subtle differences in temperature profiles among materials such as graphene and borophene can be attributed to their differing lattice symmetries and bonding strengths. In contrast, variations in BC_6_N arise from compositional differences.

Together, these findings offer a comprehensive mechanistic framework for understanding thermal-shock resistance in 2D materials. Wave velocity primarily depends on harmonic stiffness, which is influenced by bond strength, network symmetry, and areal density; hexagonal lattices exhibit the fastest, most isotropic propagation. Conversely, wave attenuation is governed by anharmonic and topological effects, such as bond-angle distortions, heteroatom-induced scattering, non-hexagonal rings, flexural-mode coupling, and localized vibrational states. Materials like graphene and C_3_N effectively minimize these anharmonic channels, resulting in high wave speeds and minimal decay, whereas borophene, biphenylene, and BC_3_ contain scattering centers that rapidly dissipate energy. Analyzing these mechanisms across various 2D structures explains why speed and damping are uncorrelated and why materials with similar elastic stiffness can have markedly different shock resistance. This mechanistic understanding provides a solid foundation for predicting and comparing the transient thermomechanical responses of emerging 2D materials.

Preliminary simulations at 100 K and 1000 K on graphene and h-BN showed that stress-wave propagation, decay, and material trends are consistent, indicating robustness of the study's conclusions. The 1000 K stress waves have sharper fronts, clearer peaks, and lower noise, thereby aiding wave tracking and decay analysis without altering core physics. Thus, 1000 K was selected for detailed analysis, with a comparison in Fig. 4S of the SI.

## 4 Conclusion

This investigation provides a comprehensive assessment of thermal-shock behavior across a wide range of two-dimensional (2D) materials, using molecular dynamics (MD) simulations to elucidate stress-wave propagation, anisotropic mechanical response, and energy dissipation at the atomic scale. By creating an abrupt thermal gradient and monitoring the evolution of longitudinal and shear stress components, atomic movements, and temperature profiles, this study captures the intricate relationship between material structure and transient thermomechanical phenomena.

The findings indicate that the response of 2D materials to thermal shock is material-specific and varies with the direction of the shock. Graphene and C_3_N, noted for their strong covalent networks and high in-plane stiffness, exhibit excellent wave transmission, minimal energy loss, and low deformation, making them ideal candidates for high-performance thermal interface materials and nanoelectronic devices. Remarkably, C_3_N exhibits undamped stress wave propagation with a decay rate of zero, indicating outstanding thermal and mechanical stability.

Borophene exhibits anisotropy, with armchair propagation faster and more attenuated than zigzag, underscoring the need for direction-sensitive analyses when designing devices containing borophene components. BC_6_N polymorphs, with intermediate wave speeds and varying decay rates, further emphasize the impact of subtle structural differences on thermal-shock resilience.

Conversely, biphenylene and BC_3_ show subpar performance under thermal stress, characterized by high decay rates, low wave speeds, and sporadic stress distributions. These characteristics indicate limited capacity to withstand rapid thermal fluctuations, suggesting that their use should be restricted to applications with minimal thermal transients or to conditions that are effectively managed externally.

Displacement analysis reveals the deformation characteristics induced by thermal shock, ranging from expansion in thermally stable materials, such as C_3_N, to contraction and potential out-of-plane buckling in more flexible lattices, such as borophene. Temperature distribution images further support these observations, showing sharp gradients and rapid thermal decay in materials with high damping.

By combining stress, displacement, and thermal data, this study presents a cohesive framework for assessing the transient response of 2D materials under extreme conditions. The decay rate is a crucial metric that reflects a material's ability to mitigate mechanical disturbances, which is essential for forecasting structural failure, fatigue, and performance in thermally stressed environments.

Ultimately, this research delivers key insights for the strategic selection and engineering of 2D materials in applications ranging from flexible electronics and thermal coatings to nanoelectromechanical systems (NEMS). It also establishes a solid foundation for future investigations into the effects of defects, multilayer interfaces, and hybrid heterostructures on thermal-shock behavior in atomically thin materials.

## Conflicts of interest

There are no conflicts to declare.

## Supplementary Material

RA-016-D5RA09164K-s001

## Data Availability

The datasets used and/or analyzed during the current study available from the corresponding author on reasonable request. Supplementary information (SI): additional tables and figures that provide further clarification and deeper analysis of the obtained results. In addition, a video file presenting the complete molecular dynamics simulations is included to better illustrate the evolution of the stress waves and thermomechanical response of the investigated two‑dimensional materials. See DOI: https://doi.org/10.1039/d5ra09164k.
