# Supplementary material for: Thermal shock resistance of various two-dimensional materials: a comparative analysis
Source: RSC Adv. 2026 Mar 23;16(18):16088–104. doi: 10.1039/d5ra09164k (PMC13006883; doi:10.1039/d5ra09164k)
Supplement: RA-016-D5RA09164K-s001 [file RA-016-D5RA09164K-s001.pdf]

Supplementary Information (SI) for RSC Advances.
This journal is © The Royal Society of Chemistry 2026

Supplementary Materials for:

Thermal Shock Resistance of Various Two-Dimensional Materials: A Comparative Analysis

Ali Ghavipanjeh[1], Sadegh Sadeghzadeh[2*], Nader Malih[3]

1- Research assistant in Nanotechnology Engineering, Smart Micro/Nano Electromechanical Systems (SMNEMS) Laboratory, School of Advanced Technologies, Iran University of Science and Technology, Tehran, Iran

2,*- Associate Professor of Nanotechnology Engineering, Smart Micro/Nano Electromechanical Systems (SMNEMS) Laboratory, School of Advanced Technologies, Iran University of Science and Technology, Tehran, Iran, Postal Code: 16846-13114.

3- Department of Physics, Faculty of Science, University of Kurdistan, 66177-15175, Sanandaj, Kurdistan, Iran

* sadeghzadeh@iust.ac.ir

*Corresponding author
[1]Email corresponding authors: Sadeghzadeh@iust.ac.ir
Tel: +982173225812

Supplementary tables

Table S1. The Tersoff potential parameters[1]

Parameter	Carbon (C)	Boron (B)	Nitrogen (N)
A (eV)	1393.6	1393.6	1830.8
B (eV)	346.7	3.467	471.18
λ_1 (Å^{-1})	3.4879	2.2119	3.2394
λ_2 (Å^{-1})	2.2119	1.5	1.3258
λ_3 (Å^{-1})	0.0	0.0	0.0
β	1.5724×10^{-7}	1.15×10^{-6}	1.178×10^{-7}
n	0.72751	0.8	0.83134
c	38049	33000	130000
d	4.3484	3.5	16.217
h	-0.57058	-0.3	-0.59825
R (Å)	1.8	2.0	1.95
D (Å)	0.2	0.2	0.15

Table S2. The Stillinger–Weber (SW) potentiel parameters for Boron[2]

Parameter	Value
ε	2.4583
σ	2.62
a	2.0
λ	31.5
γ	1.2
$\cos(\theta_0)$	-1/3 (≈ -0.3333)
A	7.049556277
B	0.6022245584
p	4.0
q	0.0

Table S3. Simulation Cell Size and number of particles

**Structure name or molecular formula**	**Simulation Boxes Dimension ($\mathring{A}^3$)**		**Particles number**	
	Zigzag	Armchair	Zigza g	Armchair
Borophene(B)	365.4*25.82*110	367.2*28.6*110	4095	4560
Biphenylene(C)	367.6*27.9*15	374.7*26.6*15	3491	3401
BC_3	363.5*26.7*15	363.2*25.7*15	3407	3279
BN	363.2*25.9*100	366.0*27.4*100	3503	3739
Graphene(C)	359.2*25.1*20	369.3*27.4*100	3575	3783
C_3N	381.0*25.8*100	377.0*29.8*14	3695	4223
BC_6N(a)	372.3*26.2*15	374.2*25.25*15	3551	3439
BC_6N(b)	370.7*26.15*15	373.2*25.13*15	3551	3439
BC_6N(c)	372.7*26.2*15	374.2*25.2*3.1	3551	3439

Supplementary figures

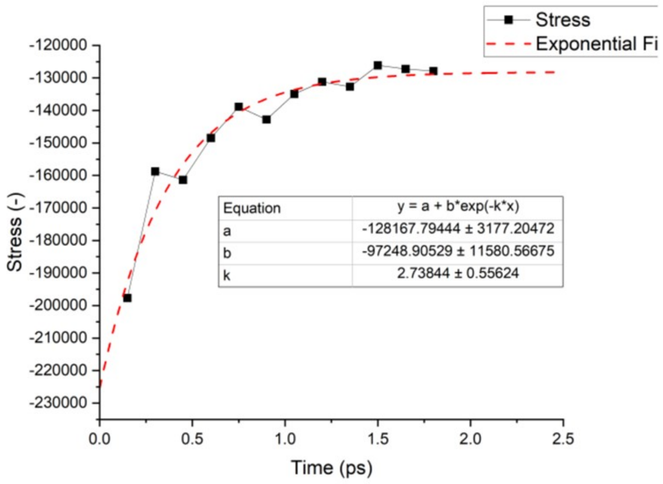

Figure S1. Fitting the stress peaks of zigzag Graphene to equation 1.

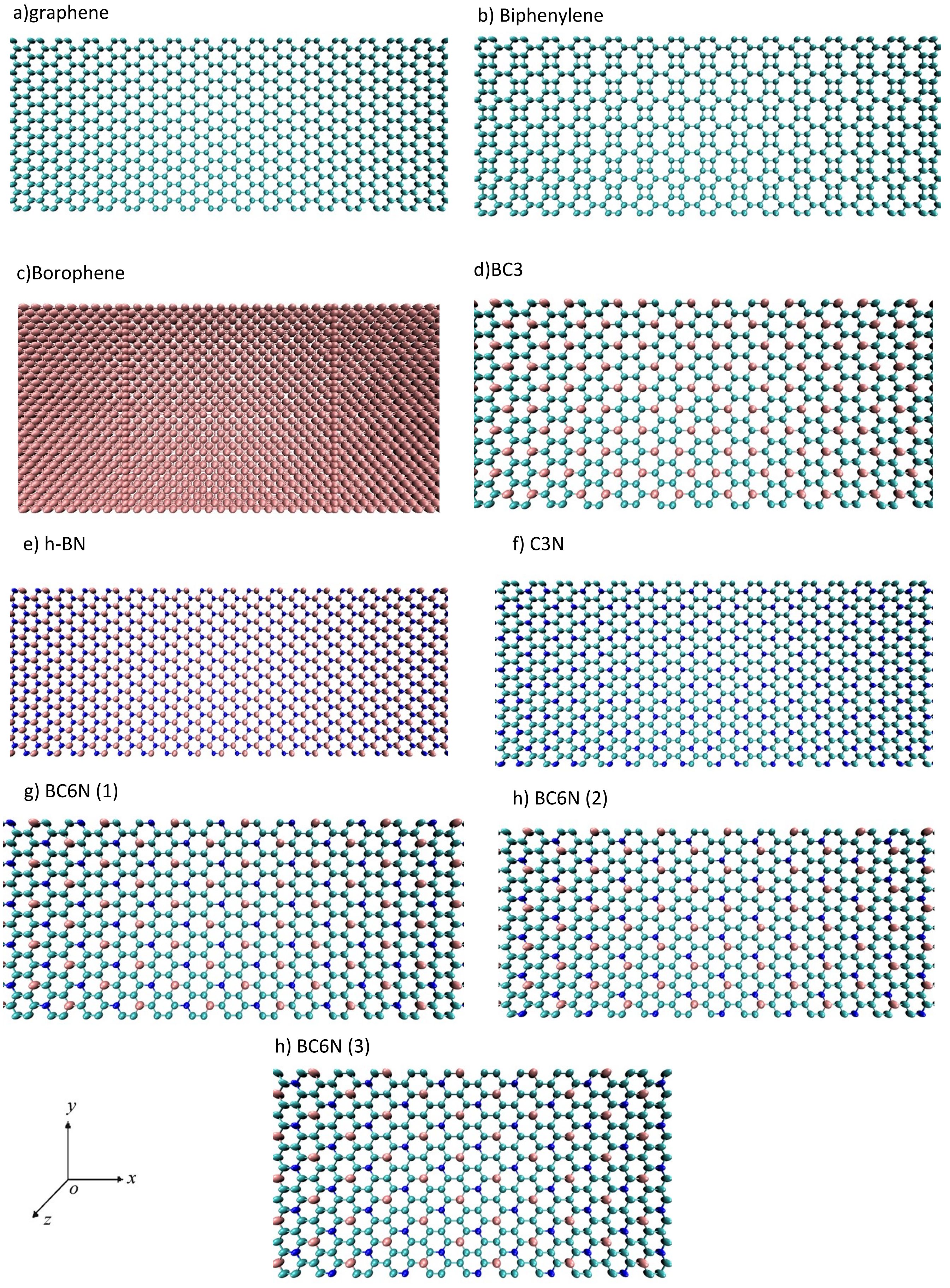
a)graphene
b) Biphenylene
c)Borophene
d)BC3
e) h-BN
f) C3N
g) BC6N (1)
h) BC6N (2)
h) BC6N (3)
y
x
o
z

Figure S2. The initial structure of the investigated 2D materials in this work(armchair orientation). a)graphene,b)biphenylene,c)borophene,d)BC3,e)h-BN,f)C3N,g,h, i) BC6N in 3 different structures. (cyan atoms as Carbon, pink as Boron, blue as Nitrogen)

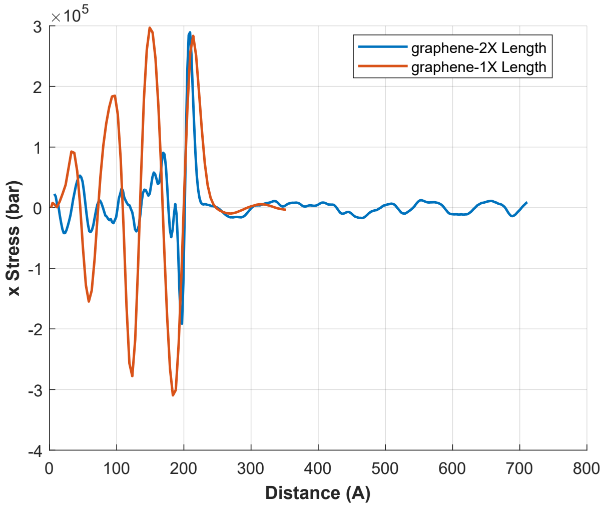

Figure S3. Effect of the Length of the Simulation Cell (in this case, Graphene) on the longitudinal Wave Direction.

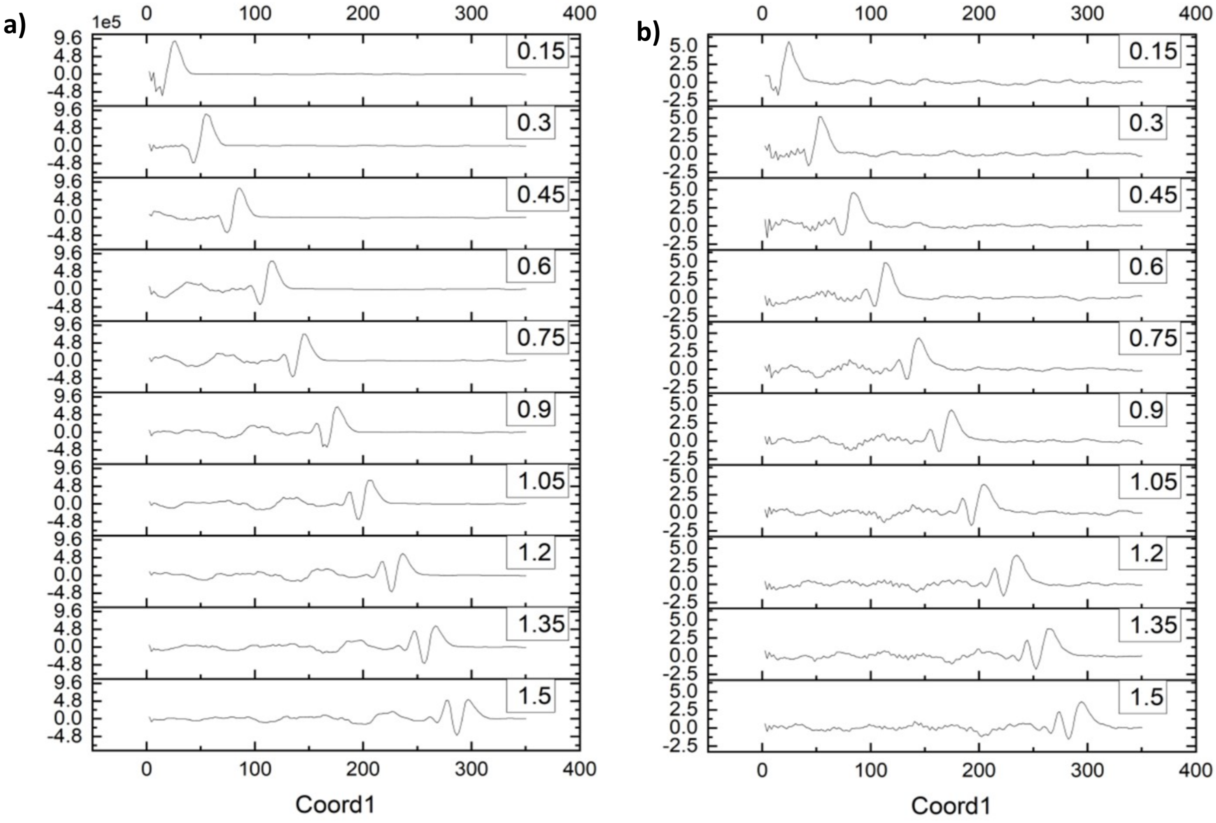

Figure S4. Effect of the two temperatures on the peak wave (in this case, h-BN) regarding the longitudinal wave direction: a) 1000 K and b) 100 K.

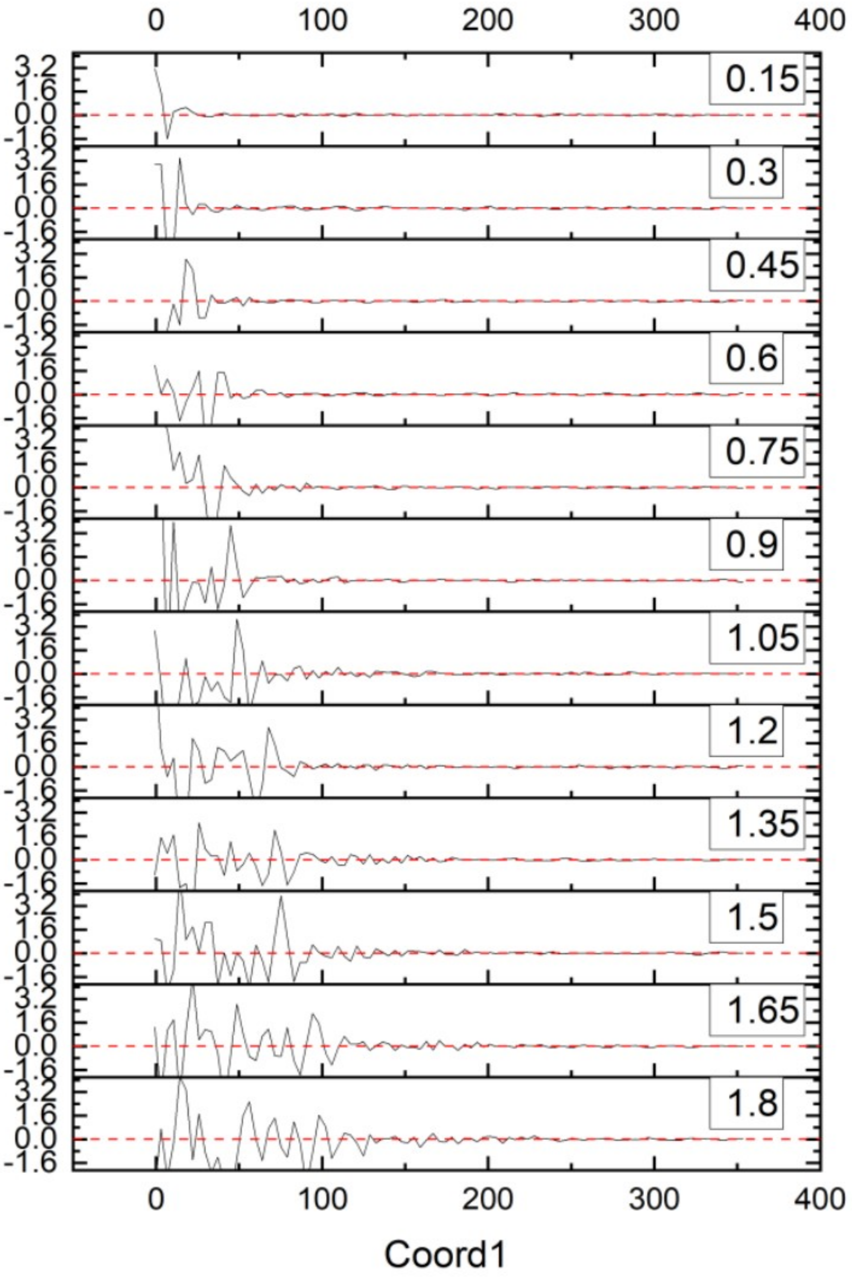

Figure S5. Shear stress (xy) in biphenylene over different times.

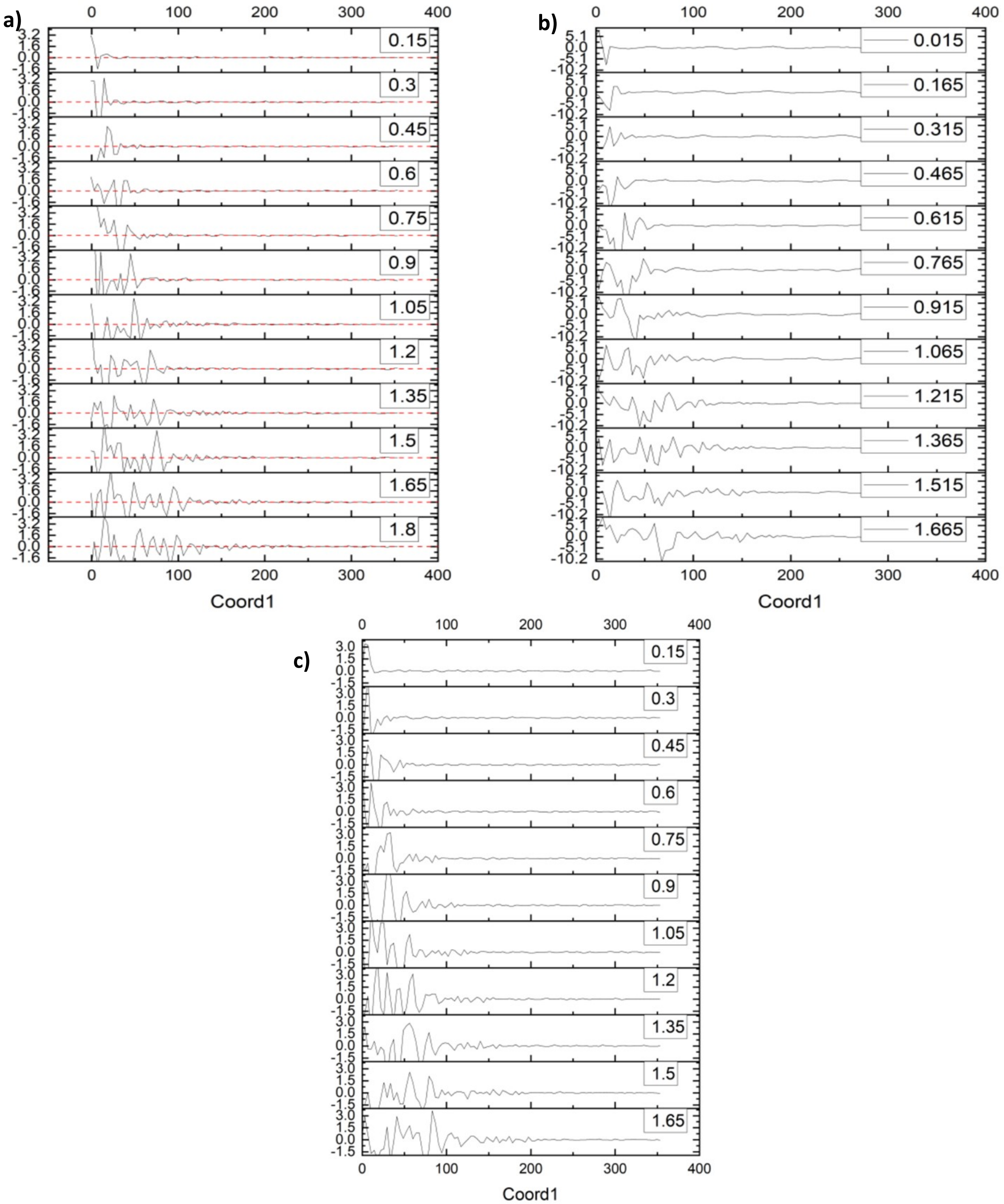

Figure S6. Shear stress wave diagram for the biphenylene structure at different times. A) Prototype with a time step of 0.5 fs, B) A time step of 0.05 fs, which is ten

times smaller than the original sample, and C) A repeated experiment with newly randomized initial velocities.

Supplementary movie

Movie S1 includes longitudinal and shear stress colour plots and stress coordination plot animations for both armchair and zigzag directions, as well as an atomic displacement plot for the investigated materials.

References

1. Kınacı, A., et al., *Thermal conductivity of BN-C nanostructures.* Physical Review B—Condensed Matter and Materials Physics, 2012. **86**(11): p. 115410.
2. Moon, W.H. and H.J. Hwang, *A modified Stillinger–Weber empirical potential for boron nitride.* Applied surface science, 2005. **239**(3-4): p. 376–380.
